# Diagnostic value of mammography density of breast masses by using deep learning

**DOI:** 10.3389/fonc.2023.1110657

**Published:** 2023-06-02

**Authors:** Qian-qian Chen, Shu-ting Lin, Jia-yi Ye, Yun-fei Tong, Shu Lin, Si-qing Cai

**Affiliations:** ^1^ Department of Radiology, The Second Affiliated Hospital of Fujian Medical University, Quanzhou, Fujian, China; ^2^ Shanghai Yanghe Huajian Artificial Intelligence Technology Co. Ltd., Shanghai, China; ^3^ Centre of Neurological and Metabolic Research, The Second Affiliated Hospital of Fujian Medical University, Quanzhou, Fujian, China; ^4^ Department of Neuroendocrinology, Group of Neuroendocrinology, Garvan Institute of Medical Research, Sydney, Australia

**Keywords:** mammographic density, deep learning model, convolutional neural network, regions of interest, breast mass

## Abstract

**Objective:**

In order to explore the relationship between mammographic density of breast mass and its surrounding area and benign or malignant breast, this paper proposes a deep learning model based on C2FTrans to diagnose the breast mass using mammographic density.

**Methods:**

This retrospective study included patients who underwent mammographic and pathological examination. Two physicians manually depicted the lesion edges and used a computer to automatically extend and segment the peripheral areas of the lesion (0, 1, 3, and 5 mm, including the lesion). We then obtained the mammary glands’ density and the different regions of interest (ROI). A diagnostic model for breast mass lesions based on C2FTrans was constructed based on a 7: 3 ratio between the training and testing sets. Finally, receiver operating characteristic (ROC) curves were plotted. Model performance was assessed using the area under the ROC curve (AUC) with 95% confidence intervals (*CI*), sensitivity, and specificity.

**Results:**

In total, 401 lesions (158 benign and 243 malignant) were included in this study. The probability of breast cancer in women was positively correlated with age and mass density and negatively correlated with breast gland classification. The largest correlation was observed for age (r = 0.47). Among all models, the single mass ROI model had the highest specificity (91.8%) with an AUC = 0.823 and the perifocal 5mm ROI model had the highest sensitivity (86.9%) with an AUC = 0.855. In addition, by combining the cephalocaudal and mediolateral oblique views of the perifocal 5 mm ROI model, we obtained the highest AUC (AUC = 0.877 P < 0.001).

**Conclusions:**

Deep learning model of mammographic density can better distinguish benign and malignant mass-type lesions in digital mammography images and may become an auxiliary diagnostic tool for radiologists in the future.

## Introduction

In 2020, WHO reported that female breast cancer had surpassed lung cancer as one of the most common cancers and leading causes of cancer deaths in the world (1). According to the GLOBOCAN 2020 database, female breast cancer prevalence and mortality will increase in the next 20 years due to population growth and ageing alone (2). In China, the incidence and mortality of female breast cancer are generally on the rise year by year (3, 4). In this regard, the “three early stages”, namely early prevention, early detection, and early treatment, are effective ways to improve the survival rate and quality of life of patients with breast cancer. Therefore, early diagnosis of breast cancer is very important.

Regular imaging screening, especially in high-risk groups, is an effective means to improve the early diagnosis rate of breast cancer. Currently, commonly used screening tests include ultrasound and radiography. Mammography (MG) is a screening method that has been proven to reduce breast cancer mortality and is the most effective and reliable screening method for early detection and diagnosis of breast cancer (5). Although many countries have introduced mammography screening, some have not significantly improved early detection of breast cancer. The main reasons for this may include two aspects: the first is that screening X-rays are not widely used, and the second is that radiologists do not accurately diagnose images. Breast mass is a most common X-ray manifestation of breast lesions. Breast mass density provides another observation for the identification of benign and malignant masses, and studies have found the degree of malignancy of the breast tumor is closely related to the density of the mass (6, 7). Nowadays, the current evaluation of mass density is mainly based on the empirical qualitative judgment of doctors, and its accuracy is limited by experience and subjectivity. How to detect the density of lesions more objectively and accurately is very important for the diagnosis of benign and malignant lesions. Therefore, our study aims to explore whether we can use artificial intelligence (AI) to quantitatively evaluate focal density.

In the past decade, with the development of AI technology, deep learning methods have been applied in medical imaging technology and have improved the accuracy of detection and diagnosis. This has helped radiologists to minimize the rate of false positives and false negatives in clinical diagnosis. Deep learning, a subset of AI, trains large-scale data by building multi-layered machine learning models, and then obtains a large amount of meaningful feature information, which is finally used to classify and predict sample data. Convolutional neural networks (CNN) are the most popular architecture for medical image analysis based on deep learning. CNNs improve the ability to accurately identify images by processing them through multiple sequential stages and representation layers and then decomposing spatially relevant information from the images into more abstract and simpler information (8).

Therefore, deep learning, especially CNN, has rapidly become the preferred method for medical image analysis. In the last five years, the computational AI revolution, mainly driven by deep learning and CNN, has also penetrated the field of automatic breast cancer detection in mammography, contributing to the early detection of diseases such as breast cancer and improving prognosis and survival percentage (9, 10). Some related studies have shown that the detection accuracy of CNN models is higher than that of computer aided system (CAD) models. Moreover, CNN models can help radiologists provide a more accurate diagnosis by quantitatively analyzing suspicious lesions (11, 12). Deep learning plays an increasingly important role in breast imaging diagnosis. Currently, the development and application of computer and artificial intelligence technologies have made it possible to quantitatively assess breast gland density. Several computer software is available to automatically measure mammographic density, such as Quantra and LIBRA (13, 14). Each of this software can obtain the percentage of mammographic density of the entire breast gland. However, reports exploring the mammographic density of any region of interest (ROI) of the breast are still scarce. Therefore, this study attempts to obtain quantitative mammographic density of ROIs of the breast masses with the help of deep learning.

Previous studies have not explored the mammographic density of breast mass lesions to any extent, and there is no software available to calculate mammographic density for any ROI. In addition, it is well known that the tumor microenvironment plays an important role in tumor growth and invasion (15, 16), and peritumor tissues have been shown to provide useful information for diagnosis and prediction of prognosis. However, how to identify and evaluate the peritumor tissues has not been systematically investigated. Therefore, we propose a deep learning model based on ROI density of breast lesions, using a novel architecture—C2FTrans (17), to quantitatively analyze the value of ROI density in the classification and diagnosis of breast masses and different regions around them for benign and malignant breast diseases. The C2FTrans, proposed by Lin, has better performance, faster speed, and greater robustness relative to the state-of-the-art CNN-based and Transformer-based approaches. The algorithm has the following advantages. First, the transformer network with sufficient receptive field and adaptive surface segmentation can effectively solve the imbalance problem in the data. Second, the local boundary network can accurately locate the boundary of the tumor. Third, the coding and decoding structure can adapt to the different shapes and sizes of tumor lesions.

By analyzing the clinical diagnostic value of the density of breast lumps, and different areas around them, in differentiating benign and malignant breast lesions, the methodology is expected to provide a more accurate and objective basis for early diagnosis and screening of breast cancer in the future, assisting physicians to work more efficiently and effectively guiding the establishment of personalized treatment plans to improve prognosis.

## Materials and methods

This retrospective study was approved by the Ethics Committee of the Second Affiliated Hospital of Fujian Medical University, which waived the requirement for individual informed consent.

### Study participants

**Figure 1:**
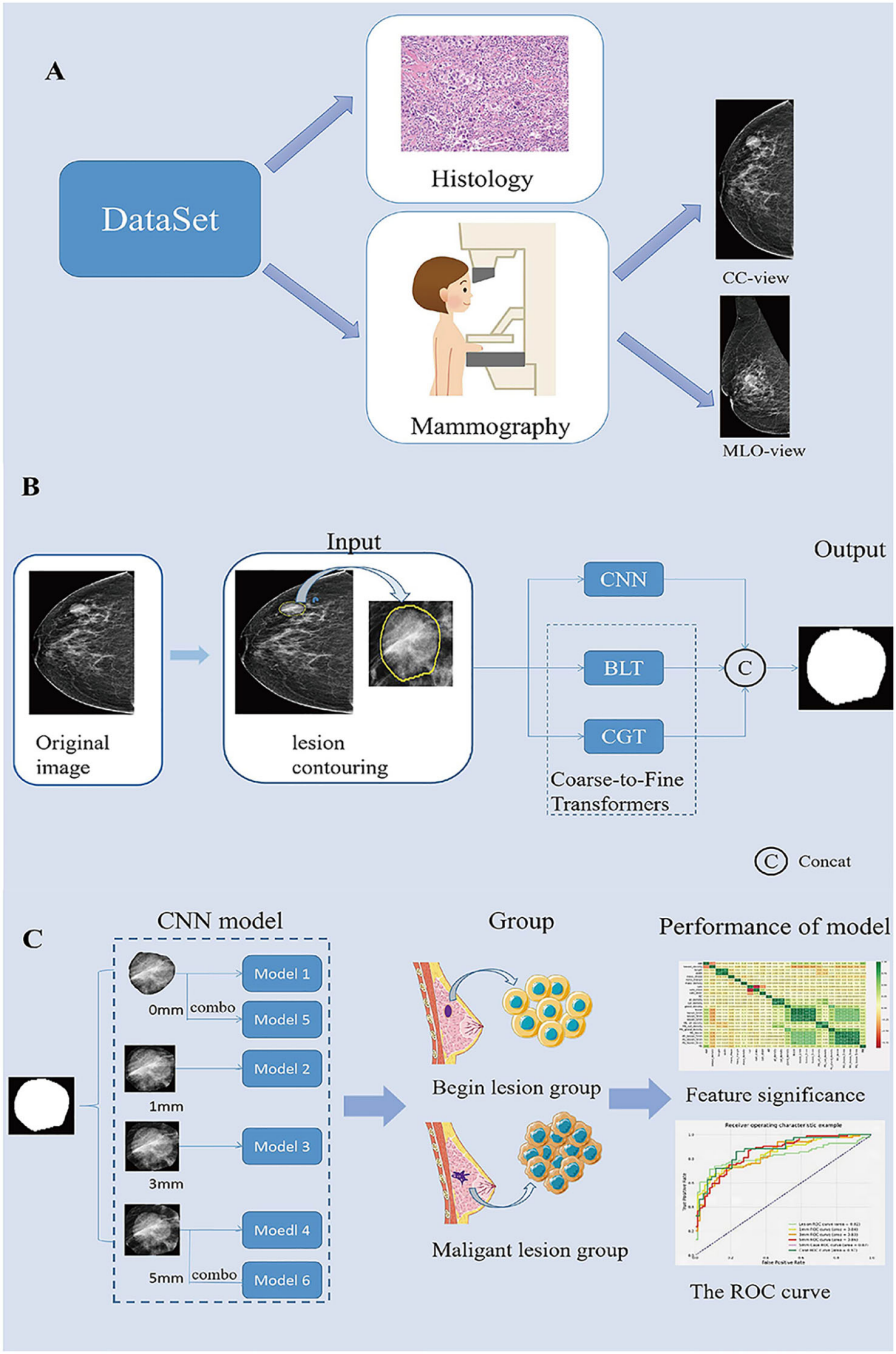
Summary of the Study. **(A)**. Description of the dataset used in this study. **(B)**. Generating process of the proposed multi-scale medical image segmentation framework based on coarse-to-fine transformers(C2FTrans). **(C)**. CNN model construction and model performance analysis. CC, cephalocaudal; CNN, Convolutional neural networks; BLT, Boundary-aware Glocal Transformer; CGT, Cross-scale Global Transformer; MLO, Medio-lateral oblique; ROC, The receiver operating characteristic.

We retrospectively analyzed the profiles of female patients who underwent histological biopsy and mammography at the Second Affiliated Hospital of Fujian Medical University from August 2016 to December 2021. In all cases, the underlying lesion on Full Field Digital Mammography (FFDM) images appeared as masses, and the lesions were not accompanied by other underlying lesions e.g., calcification or structural distortion. We excluded patients based on the following criteria: (1) poor image quality; and (2) a history of breast surgery, breast radiotherapy, chemotherapy, or hormone therapy (**Figure 1A**).

### Image analysis

For each patient, cephalocaudal (CC) and medio-lateral oblique (MLO) views of both breasts were obtained. All mammograms were reviewed by two radiologists. The classification of the mammary glands, lump sizes and shapes, and the margins of the breast masses were evaluated with reference to the Breast Imaging Reporting and Data System. The maximum diameter of the lesion was measured independently by two radiologists at the workstation, and the average of the two maximum values was taken as the final lesion size. In case of disagreement, the final decision was made by consensus.

### Image segmentation

The image segmentation process included manual segmentation of the lesion and automatic segmentation of the surrounding area. All segmentation steps were performed in the CC and MLO views of the images in Digital Imaging and Communications in Medicine format. First, Radiologist 1 (with 5 years of experience in breast work) examined the mammographic images and manually contoured the lesions using 3D-Slicer (Version: 4.11.20210226 ;www.slicer.org). All contouring results were reviewed and agreed upon byRadiologist 2, who has more than 10 years of experience in the field of breast cancer. Then, the lesion and its surrounding area with 0-, 1-, 3-, and 5 mm (including the lesion area) were automatically segmented using the C2FTrans segmentation framework (**Figure 1B**). Finally, the density of the breast, the ROI density of the mass, and the density of different areas around the mass were obtained, and a diagnostic model of the breast mass was constructed according to the ratio of 7:3 between the training set and the testing set.

### Deep learning models training and testing

In this study, we used C2FTrans (17), a multi-scale segmentation framework based on coarse-to-fine transformers that can be used to segment medical images of different shapes and sizes as a coarse-to-fine process. C2FTrans consists mainly of a cross-scale global transformer and a boundary-aware local transformer. The former deals with local context similarity in CNNs, and the latter overcomes the boundary uncertainty associated with rigid division in transformer, thus reducing the computational complexity and detail loss based on large-scale feature mapping. U-Net and U-Net ++ were selected as comparison models to conduct experiments on the FFDM image data set of breast mass, respectively, to verify the segmentation effect of this method (C2FTrans), compared with the typical methods in the field of medical image segmentation in recent years.

Based on the obtained mass ROI density (Model 1) and mammographic densities of the mass and different surrounding areas (Models 2-4), we constructed the corresponding learned diagnostic models (as shown in the model diagram). In addition, we combined the CC and MLO views of the mass ROI and the surrounding 5-mm area to construct case 0 (Model 5) and case 5 (Model 6), respectively (**Figure 1C**). Sensitivity, specificity and area under the curve (AUC) were used to evaluate model performance. Sensitivity is the probability that the model output is positive (malignant) when the sample is malignant; specificity is the probability that a given sample is benign when the model output is negative(benign); and AUC is the average sensitivity of all possible specificity values.

### Histological analysis

The references were the histopathological diagnoses obtained by biopsy or surgery after mammography. Malignant cases were defined as lesions or ductal carcinoma *in situ* with an invasive component. Benign lesions were defined as lesions or carcinoma *in situ* without any invasive components.

### Statistical analysis

This study used Dice Similarity Coefficient (DSC), Recall (equal to sensitivity), and Intersection over Union. Objective evaluation indicators such as IoU specificity were used to evaluate the performance of specificity for breast mass segmentation.

All statistical analyses were performed using SPSS 26.0 software. Continuous variables were expressed as mean ± standard deviation and differences were compared using the independent samples t-test for normal distribution and the nonparametric Mann-Whitney U test for non-normal distribution. Categorical variables were expressed as percentages (%) and differences were compared by rank sum test. The deep learning model was evaluated for diagnostic performance by plotting the ROC curve and analyzing the AUC with 95% confidence intervals (*CI*), specificity, and sensitivity. A two-sided P value of less than 0.05 was considered statistically significant.

## Results

### Data sets

A total of 401 lesions were included in this retrospective study, including 158 benign lesions and 243 malignant lesions. The general clinical characteristics of the subjects in this study are summarized in **Table 1**. In the training and test sets, there were significant differences between the benign and malignant groups in terms of age, glandular classification, and mass density (*P*<0.001). However, the size of the mass lesion was not statistically significant for benign and malignant breast mass lesion classification (*P*=0.303).

**Table 1:** General clinical characteristics of the study subjects.

	Totality (n=401)	Benign (n=158)	Malignance (n=243)	P value
Age, y	47.0 ± 11.7 (100%)	40.2 ± 10.7 (33.7%)	51.4 ± 10.2 (66.3%)	<0.001***
ACR category				<0.001***
a	10(2.5%)	0(0)	10(4.1%)	
b	37(9.2%)	4(2.5%)	33(13.6%)	
c	315(78.6%)	134(84.8%)	181(74.5%)	
d	39(9.7%)	20(12.7%)	19(7.8%)	
Maximum diameter of the mass, cm		2.0(1.8-2.5)	2.1(1.7-2.7)	0.303
CC-mass density	2023.2 ± 19.4	1907.0 ± 31.0	2098.7 ± 23.6	<0.001***
MLO-mass density	2059.7 ± 17.9	1939.4 ± 30.1	2138.0 ± 20.7	<0.001***

***Mean statistically significant difference on test.

ACR, American College of Radiology. a. Breasts are almost entirely fat (about 10% of women). b. A small amount of breast tissue is scattered in the breast (about 40% of women). c. Mammary glands are evenly distributed throughout the breast (approximately 40% of women). d. Very dense breasts (about 10% of women). CC, The craniocaudal projection. MLO, The mediolateral oblique projection.

**Figure 2:**
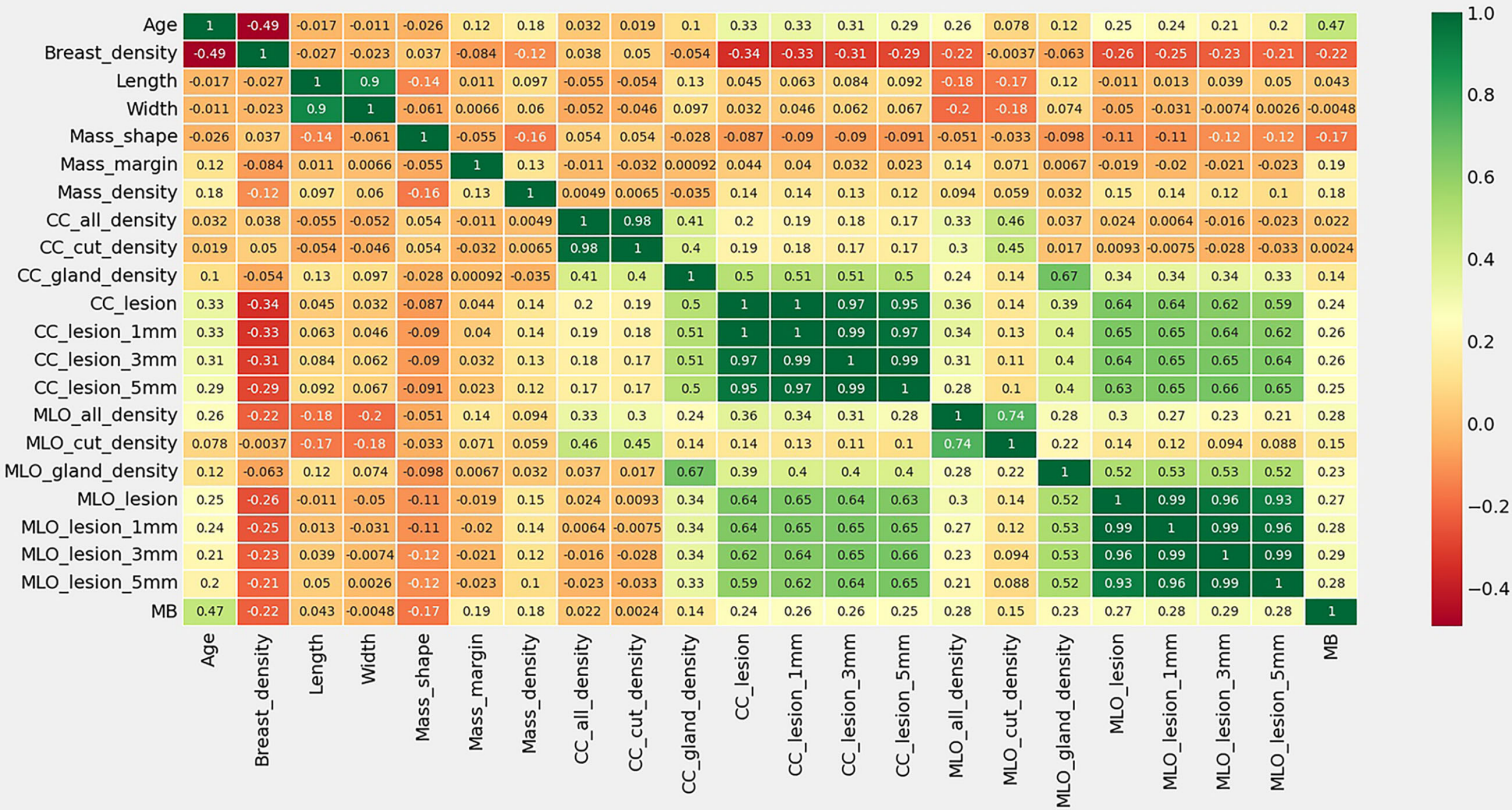
Thermal spectrum of correlation coefficient of characteristic statistics of research subjects. MB means the pathological findings of Malignant or Benign lesions.

**Figure 3:**
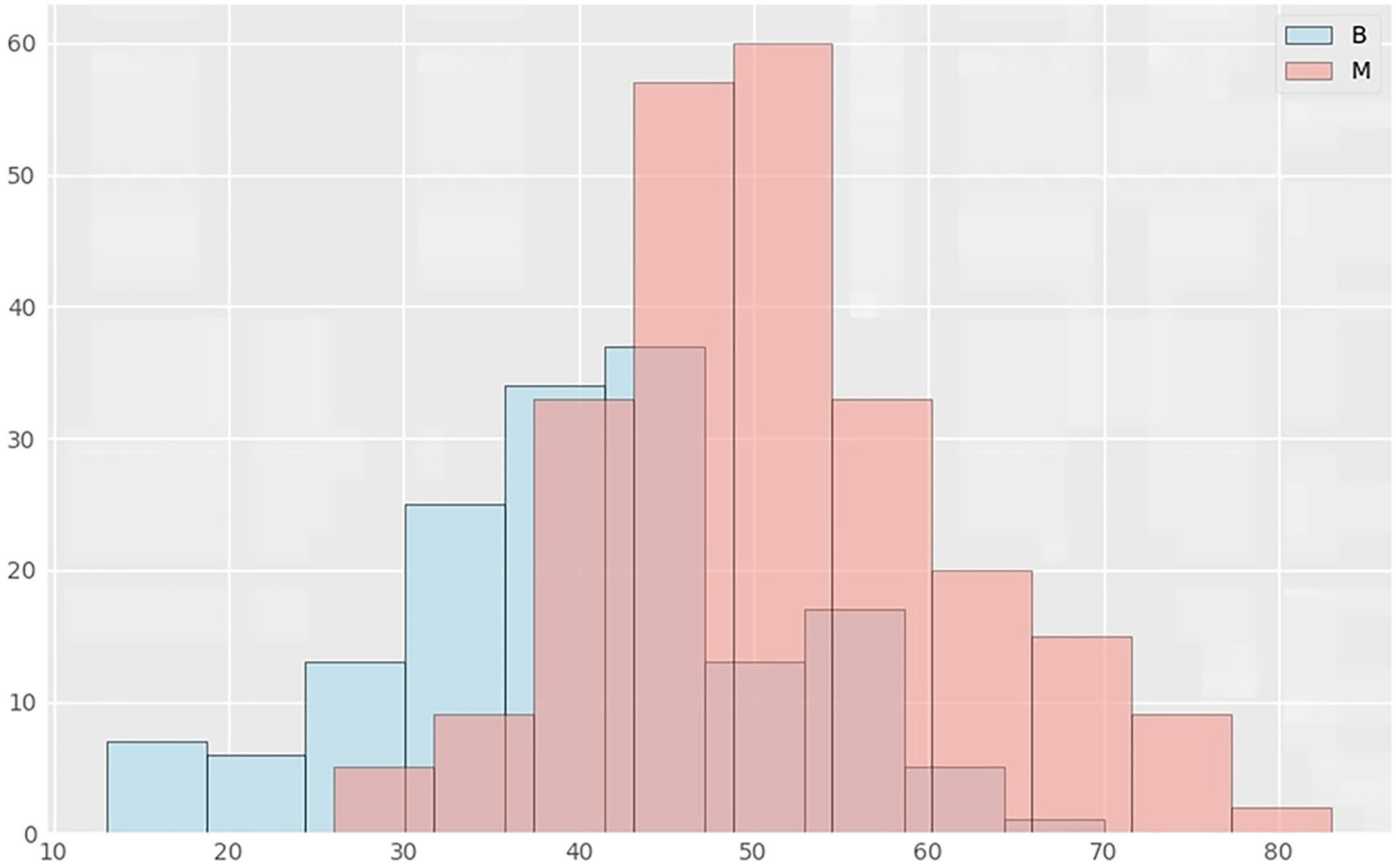
Distribution histograms of age and lesions in benign and malignant groups. The abscissa in the histogram represents age, and the ordinate is the number of cases in the corresponding age group. B represents the benign lesion group; M represents the malignant lesion group.

Among the variables we counted: age, breast gland type, long and wide sides of breast lump lesions, mass shape, mass margin characteristics, mass density, breast density of any ROI, and benign and malignant mass lesions. According to the thermal spectrum (**Figure 2**), benign and malignant breast lesions were positively correlated with age, mass density and glandular density. There was a negative correlation with breast gland typing. Among them, the age-related coefficient was the highest(r=0.47). Therefore, histograms of age and the number of benign and malignant lesions, were plotted (**Figure 3**). The results clearly showed that malignant cases mostly occurred between the ages of 43 and 55, while benign cases usually occurred between the ages of 30 and 47.

### Performance of the deep learning models

**Table 2** shows the results of comparison evaluation indexes of C2FTrans model proposed in this paper and other methods. DSC, Recall, IoU and specificity were all above 0.85, and the specificity was close to 1, indicating the low misdiagnosis rate of this model. The DSC, Recall and IoU of C2FTrans model were all higher than those of U-Net and U-net ++, suggesting that the C2FTrans model proposed in this study has better segmentation performance.

**Table 2:** Comparison of objective evaluation indicators between different segmented networks.

model	Testing				Training			
	DSC	recall	IoU	Specificity%	DSC	recall	IoU	Specificity%
UNet	0.744	0.744	0.715	99.9	0.843	0.826	0.794	99.9
UNet++	0.768	0.876	0.691	99.6	0.882	0.873	0.855	99.9
C2FTrans	0.889	0.913	0.850	99.7	0.902	0.932	0.887	99.8

**Figure 4:**
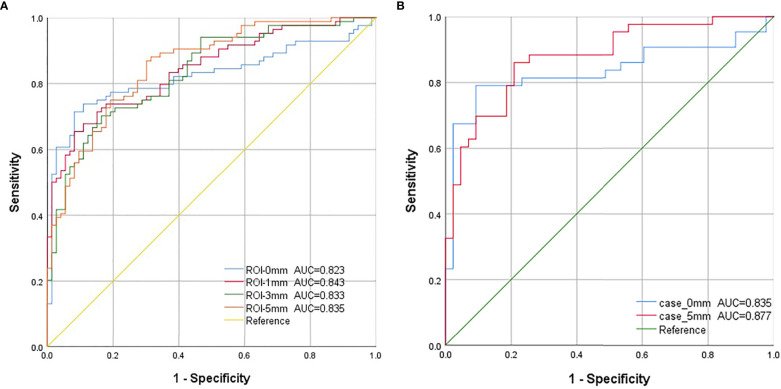
The ROC curves for each model in this study. **(A)**. Model 1-4: The blue curve is the receiver operating characteristic (ROC) curve of the model (model 1) constructed using features extracted from the mass region of interest (ROI). The red curve represents the ROC curve of the model (model 2), which was constructed using image features extracted from the ROI around the 1 mm lesion. The green curve represents the ROC curve of the model (model 3), which was constructed using the image features extracted from the ROI around the 3 mm lesion. The orange curve is the ROC curve of the model (model 4), which was constructed using image features extracted from the ROI around the 5-mm lesion. **(B)**. Model 5-6: Combining both cephalocaudal and medio-lateral oblique dual views. The blue curve is the model constructed using image features extracted from the tumor ROI after combining the dual views (model 5), and the red curve is the model constructed using image features extracted from the 5mm lesion surrounding ROI after combining the dual views (model 6).

Our deep learning-based mammography density diagnostic models demonstrated good diagnostic performance, and the AUCs of all the models were greater than 0.800. The classification and diagnostic performance results of the mammography density models constructed by deep learning in the study are shown in **Table 3**. The ROC curves of each model are shown in **Figure 4**. Among all our models, the mass ROI model alone (Model 1) had the highest specificity (91.8%), and the 5 mm ROI model around the mass (Model 4) had the highest sensitivity (86.9%) compared to other models (**Figure 4A**). Based on the above results and combined with the daily work experience of radiologists, we selected a subset of cases in the dataset with both CC and MLO views. We combined the two body views to construct Case-0 and Case-5, respectively (**Figure 4B**). We found that the case AUC of the combined two body image data was higher than that of the single position image (AUC _Model 5 = _0.835 vs AUC _Model 1 = _0.823;AUC _Model 6 = _0.877 vs AUC _Model 4 = _0.855), and the AUC of the Case-5 model (model 6) could reach 0.877 (95% CI: 0.805-0.949 *P*<0.001).

**Table 3:** The performance of the deep learning models.

Deep Learning models	AUC(95%CI)	Sensitivity, %	Specificity, %
Model 1:mass alone (ROI-0)	0.823(0.754~0.892)	71.4	91.8
Model 2:mass+perilesional ROI-1mm	0.843(0.784~0.903)	71.4	84.9
Model 3:mass+perilesional ROI -3mm	0.833(0.770~0.895)	70.2	83.6
Model 4:mass+perilesional ROI -5mm	0.855(0.798~0.912)	86.9	69.9
Model 5:case-0	0.835(0.740~0.930)	79.1	90.7
Model 6:case-5	0.877(0.805~0.949)	86	79.1

Case: A patient’s mammograms with both cephalocaudal and medio-lateral oblique views.

AUC, area under the receiver operating characteristic curve; CI, confidence interval; ROI, region of interest.

## Discussion

In this study, six deep learning-based mammographic density diagnostic models were proposed and their diagnostic performance was evaluated. We have summarized the technical route and some of the results of this study in **Figure 1**. Key findings are: 1) Age had a high positive correlation in benign and malignant classification of breast lesions compared with other characteristics, and age was significantly associated with breast mass lesions; 2) The mass only ROI model (Model 1) had the highest specificity (91.8%), which would help improve radiological diagnostic performance and reduce misdiagnosis of benign masses in diagnostic workup; 3) A 5-mm ROI around the masses were more valuable in differential diagnosis than other ROIs, providing more imaging information on normal tissue and reducing the missed diagnosis rate of early breast cancer screening by radiologists (4). The AUC of the model with a combined CC and MLO view could be increased to 0.877 at the highest, suggesting that the combined dual-position view might achieve more satisfactory diagnostic performance compared to image information from a single position.

From our results, it is clear that age has the highest correlation with the classification of benign and malignant breast lesions, with more women more likely to have malignant breast lesions between the ages of 43 and 55 years. This is consistent with two age peaks for breast cancer in Chinese women: one at the age of 45-55 and the other at the age of 70-75 (18). According to GLOBOCAN 2020 data, the median age of breast cancer incidence in Chinese women is 50-54 years old, earlier than that in women from Europe and the United States (60-64 years old). An increasing number of Chinese women are developing breast cancer at a younger age (19). Therefore, it is important for Chinese women to start breast cancer screening as early as possible in order to detect suspicious lesions early, improve prognosis, and reduce the burden of disease.

The detection, segmentation and classification of image lesions were achieved with the help of deep learning. Additionally, better diagnostic performance was achieved with applications in mammography, ultrasound, and magnetic resonance imaging (MRI) (20–24). This result is consistent with the deep learning model for mammography in this study. Ultrasound deep learning models were built by Fujioka and his colleagues using multiple CNN architectures, and the results showed that the deep convolutional neural network model has better diagnostic performance (20, 25). Previous studies have used different CNN approaches to classify benign and malignant mammography images with good diagnostic performance (26–28). However, there are difficulties in using deep learning for mass segmentation in mammographic images. They include problems of dataset imbalance, diversity of mass shapes and sizes, occlusion and overlap. Therefore, we utilized the CNN multi-scale architecture C2FTrans as a breast mass lesion segmentation algorithm in our study. C2FTrans is a novel multi-scale architecture developed by Lin et al. that allows medical image segmentation as a coarse-to-fine process, reducing the computational complexity and detail loss based on large-scale feature mapping. After conducting extensive experiments, C2FTrans performed better than the current state-of-the-art CNN-based and transformer-based methods. The algorithm has the following advantages. First, the transformer network with sufficient receptive field and adaptive surface segmentation can effectively solve the imbalance problem in the data. Second, the local boundary network can accurately locate the boundary of the tumor. Third, the coding and decoding structure can adapt to different shapes and sizes of tumor lesions. In the same way, the C2FTrans-based deep learning models constructed in this study also achieved better accuracy (AUC > 0.8). The single-mass ROI model had the best specificity and could accurately diagnose breast cancer.

The surrounding tissues can provide useful information for diagnosis and prognosis prediction. However, there has never been a systematic study on peritumoral tissue selection of different degrees in deep learning. Therefore, one of the aims of this study was to evaluate their diagnostic role by comparing different ranges of peritumoral tissues. With the increase of the peritumoral scope of dynamic contrast enhanced (DCE)-MRI, the diagnostic performance became worse, as comprehensively highlighted by Zhou (29). A study has compared the value of different range of radiomic features of contrast-enhanced mammography images in differentiating benign and malignant lesions. and reported the highest diagnostic performance of the AUC=0.930 in the test set with a 3mm circumferential area (30). However, the results of their study are not identical to those of ours. Our findings suggest that increasing the area around the lesion increases sensitivity but decreases specificity. The possible reason for this is that the inclusion of too much normal tissue dilutes the information of the mass, resulting in a decreased specificity of the model for mass judgment. Therefore, we can choose different areas around the tumor according to different clinical applications to diagnose the disease. For example, if our goal is to screen for breast cancer early, a 5mm peritumoral area could be selected to reduce false-negative diagnoses. If the goal is to accurately diagnose benign and malignant lesions, a separate lesion area model can be selected as an auxiliary diagnostic tool to achieve a higher level of accuracy.

In summary, we investigated the results of a single position image of the lesion. In order to be consistent with the daily work of radiologists, we selected 68 cases and combined CC and MLO image data of the lesions to construct a mass alone and a 5mm peri-tumoral area model (Case-0 and Case-5). We found that the classification diagnostic model combining CC and MLO view image information performed better than the classification diagnostic model using only CC or MLO view images. This is consistent with the previous findings (31) and with one of our earlier expectations or research hypotheses. The possible reason for this higher accuracy is that analyzing the lesion by extracting the information from only one view would lead to neglecting important features which might be only visible from the other view. Accordingly, using dual view images provide more information, which essentially leads to higher diagnostic accuracy. Therefore, it is hoped that this study can provide new scientific evidence or data for the further development of multi-view studies in the future.

Our study has several limitations. First, our study was a single-center study with samples from the same hospital, which may have led to selection bias. For future studies, it would be desirable to reduce study selection bias through the collaboration of multiple research centers. Second, the sample size of this retrospective study was relatively small. Although the performance of the constructed model was stable and the obtained results were promising, a larger prospective study is needed to validate the predictive efficiency of the model. Third, in our study, we acknowledged the high proportion of malignant breast lesions (60.6%), implying that there might have been a potential patient selection bias. Therefore, balanced datasets were also important for developing deep learning classification models. Fourth, due to the characteristics and inherent limitations of CNN and its algorithms, we extracted 2D breast density and other image features on FFDM images. Compared with 3D density and features, it might lose some lesion information. However, the results showed that the 2D-based features also displayed good performance in the classification of breast mass lesions. In the future, the model could be tried to be applied to Digital Breast Tomosynthesis to obtain the bulk density and to detect more realistic lump density.

## Conclusion

Our study found that 5mm ROI around the mass combined with CC and MLO views on DM images were more helpful in differentiating benign and malignant breast mass lesions and may improve diagnostic efficiency. Deep learning models may improve the accuracy of breast disease diagnosis in future practice, reduce the misdiagnosis of benign masses to some extent, and become an important auxiliary diagnostic tool for radiologists. Our study was only focused on the diagnosis of breast lesion classification based on masses and peri-masses and did not explore the correlation between the area around malignant masses and the invasive extent of cancer components, predicted breast cancer prognosis and lymph node metastasis. Artificial intelligence and deep learning have not been used to their full potential for breast cancer diagnosis, staging and prognosis prediction, so further research and development is still needed.

## Data availability statement

The raw data supporting the conclusions of this article will be made available by the authors, without undue reservation.

## Ethics statement

The studies involving human participants were reviewed and approved by the Ethics Committee of the Second Affiliated Hospital of Fujian Medical University. Written informed consent for participation was not required for this study in accordance with the national legislation and the institutional requirements. Written informed consent was obtained from the individual(s) for the publication of any potentially identifiable images or data included in this article.

## Author contributions

S-QC and Q-QC designed and led the present study. S-TL and J-YY reviewed the series of mammogram of those patients. Y-FT created the deep convolutional neural network of density. Q-QC and S-TL performed the statistical analysis. SL critically revised the manuscript. All authors participated in the vetting of the data and preparation of the manuscript. All authors have approved this manuscript for submission. All authors contributed to the article and approved the submitted version.
